# Identifying obstacles to participation in a questionnaire survey on widowers' grief

**DOI:** 10.1186/1472-684X-9-7

**Published:** 2010-04-29

**Authors:** Bragi Skulason, Asgeir R Helgason

**Affiliations:** 1 School of Health and Education, Reykjavík University, (Menntavegur 1) Reykjavik (IS101), Iceland; 2 Department of Public Health Sciences, Social Medicine, Karolinska Institutet, Stockholm (SE17177), Sweden

## Abstract

**Background:**

The aim of this study was to determine if Icelandic widowers might foresee obstacles to responding to a questionnaire on bereavement. Also, we sought to compare the proportion of men reporting obstacles in a telephone interview to the actual response rate in the questionnaire survey.

**Methods:**

The study was part of a nation-wide survey of widowers who lost their wives in 1999, 2000, and 2001. This included all widowers born in Iceland 1924-1969 (aged 30-75 years) who were alive, and residing in Iceland at the time of the study. A telephone poll was conducted prior to sending out a questionnaire to determine if the widowers would be interested in responding, or if they could see obstacles, which could affect their willingness to respond to a subsequent questionnaire survey. The telephone poll was repeated five years later with a random sample of the original study base to determine if views initially expressed towards the questionnaire survey, had changed over time.

**Results:**

Of the 357 eligible widowers, 11 had died prior to the first telephone interview, yielding a study population of 346 widowers. Of those, 296 (86%) were reachable and all of these (100%) were willing to participate in the telephone survey. Of them, 55% identified obstacles to participation in the questionnaire survey. Men under 60 years were less likely to identify obstacles. Years from loss (second through fourth years) were not associated with reporting obstacles to participation. The response rate in the epidemiological questionnaire survey following the telephone interview was 62% (216/346).

Of those who did identify obstacles 23%, did not did not identify any particular obstacle, but 33% stated that "they felt bad" or that it would be "a painful experience" or that they felt "uncomfortable" talking about their grief. About 18% stated their grief was "a private matter"; 6% stated that they did not want to be "stuck with their grief"; 9% said that it was "too late" to talk about their grief or that they "wanted to look towards their future". Additionally, 11% stated "other reasons", including responses like: "it's too early to talk about it", and "I have started another relationship - don't want complications."

**Conclusions:**

The willingness to participate in the telephone interview was high and indicates a strong interest in the subject. Also, exposure to the study appeared to increase willingness to participate, since many men who initially could see obstacles to participation, actually participated in the epidemiological questionnaire survey. However, approximately one third of the men who initially identified obstacles to participation remained negative toward participation throughout the study period.

## Background

There are scarce data regarding the psychological, emotional and social impact of spousal loss and its effect on long-term psychological well-being on the surviving spouse. The studies that have been reported are mainly focused on widows [1-7]. We know of only one other nation-wide epidemiological study, where psychological morbidity has been assessed, in a representative sample of all widowers [8].

In one study of widows and widowers a number of indices of health and emotional disturbance affected response rate (30%) [9]. Method of data gathering may also be of some significance. However, one study compared home interviews vs. questionnaire and found the response rate to be the same (40%) [10]. Depression may also be a factor influencing response rate [11].

The psychological well-being and bereavement of families and significant others of terminal patients are an important part of palliative care as defined by the World Health Organization (WHO) [12].

Consequently, there is a pressing need to develop research in this field.

However, a potential problem is that questionnaires assessing factors related to grief and bereavement may involve ethical complications, because some mourners may have severe emotional reactions from difficult and/or confronting questions in questionnaires.

In one study, recently widowed people were invited to participate in an interview study on loss and its effects. Any resistance to participation resulted in an apology for the intrusion and exclusion from the study. Two years later, a small sample of those who had initially declined participation were contacted by telephone and interviewed regarding their initial feelings towards the study. Most reported that they had been unwilling to talk to a stranger at a time when they were upset [13].

These results are a cause for concern when conducting a survey on bereavement. It is a logical hypothesis that certain questions may evoke negative feelings in widows and widowers and, consequently, reduce willingness to participate in bereavement studies. However, there are scarce data supporting the possible long-term, negative psychological impact of questionnaires on bereaved people. Some published research indicates participation in research may actually have a positive effect on psychological well-being in the long run [14-18], even though disclosure of emotions may [19] or may not facilitate adjustment or reduction of distress [20,21]. Studies of the role of social support in protecting the bereaved against the impact of bereavement, and helping them to cope with the loss, are inconsistent [22].

The present authors received repeated warnings from colleagues that experience in the bereavement research indicated it might be extremely difficult to obtain adequate response rate from bereaved Icelandic males. The first author had extensive experience in working within the educational and support resources of a Bereavement Association, and male participation was only 10-15% over a five- year period, compared to 85-90% female participation. It was consequently deemed risky to invest research funds in an epidemiological study on male bereavement in Iceland. Also, based on ethical considerations we wanted to be extra careful in not exposing men, who could see obstacles to participation, to the questionnaire. The logical answer was to conduct a telephone poll ahead of sending out a questionnaire, to assess and address possible obstacles of the target population to participation in an upcoming questionnaire survey, and to alert the widowers that a questionnaire would be arriving shortly.

The aim of the present study was to assess if widowers were willing to respond to a questionnaire on bereavement. We also wished to identify what obstacles (if any) the widowers mentioned for not wanting to participate in a questionnaire survey. Further, the authors wanted to compare the proportion of men reporting obstacles in the telephone interview, with the actual response rate in the upcoming survey.

Finally, we wanted to learn if attitudes towards participation in a questionnaire survey changed over time, and to what extent initial attitudes towards participation influenced the response rate. Our study was the first nation-wide survey on male bereavement in Iceland. We are not aware of any other published study in the literature where attitudes towards participation in a questionnaire survey are assessed prior to sending out a questionnaire.

## Methods

The present study is part of an extensive effort to assess bereavement among Icelandic widowers who lost their wives 1999-2001. It is the first nation-wide survey on male bereavement in Iceland.

A general census of the population in Iceland is routinely conducted at the end of every year. The total population as of December 1, 2001 was 286.575, comprising 143.450 men and 143.125 women [23].

Our present study population was defined as all widowers born 1924-1969 (aged 30-75 years at the time of selection) who had lost their wives in 1999, 2000, and 2001. Only Icelandic citizens residing in Iceland, who were still alive at the time of the study, were included in the study base, a total of 346 widowers. Identification of the study population was obtained through the Icelandic National Registry, on December 31, 2001, providing an initial total of 357 widowers, whereof 11 had died at the time of the study. Only widowers who had been formally married were included. This ensured a standard level of commitment.

The study was approved by the following official agencies:

The National Bioethics Committee of Iceland on June 28, 2006 (no. VSN200620033/03-7).

The Icelandic Data Protection Authority May 17, 2006 (no. 2006020102).

The Icelandic National Registry on November 12, 2008.

Confidentiality and anonymity were stressed as of utmost importance.

### The first telephone survey

The initial survey took place during October 26 to November 10, 2002. Traditional lunch and dinner hours were respected, and no calls were made after 21:00 hours. Up to five attempts were made to contact each widower. After that, it was assumed the widower in question was unreachable by phone.

The interview was semi-structured, according to the following protocol/script:

#### After confirmation of status as a widower

"We are planning a nation-wide study on the effects of becoming a widower. This involves this telephone poll, and a questionnaire survey which will be sent to all widowers who lost their wives 1999-2001. The questionnaire is anonymous, and, therefore, responses cannot be traced to individuals. The study has been approved by the National Bioethics Committee, the Icelandic Data Protection Authority, and the Icelandic National Registry.

In this telephone poll, we would like to ask if it is all right if we discuss the possibility of your eventual participation in a study of this kind, and to inquire if you can see any obstacles to participation. And we will respond to any questions you may have concerning the questionnaire survey. Would you be willing to talk to us briefly on the phone about this?"

#### After telephone interview consent, if given

"What is your attitude towards participation in a questionnaire on bereavement? Do you see any obstacles to participation in such a survey?"

#### If the widower sees no obstacles to participation

"Do you have any questions concerning the survey?" Any questions asked, are discussed and answered.

#### If the widower sees obstacles to participation

"Would you tell us what obstacles you see?"

#### If there was more than one obstacle

"Would you highlight the most significant one?"

At the end of the telephone interview, it was repeated to all participants that since they belonged to the cohort of Icelandic men who had become widowers during the past three years, they would receive a questionnaire by mail. It is important to emphasize they were never asked if they would participate in the questionnaire survey, which would follow the telephone poll. It was explained that they need not feel obliged to open the envelope containing the questionnaire. If they did not wish to participate, they were advised to throw away the unopened envelope, or return it to the sender. The interviews lasted 10-15 minutes. At the end of the telephone interview, we thanked the widowers for their participation in the telephone survey.

The answers regarding obstacles were categorized according to the following criteria: A) Widowers seeing no obstacles to participation; B) Widowers expressing reluctance towards participation; C) Widowers who could not be reached.

One to two weeks after the initial telephone interview, a questionnaire was sent to all 346 widowers, who were alive at the time.

### The second telephone survey

A follow-up telephone survey was conducted five years later, using a random sample of widowers from the original study base.

The main aim was to assess if the men had changed their minds regarding obstacles to participation in future bereavement questionnaire surveys. We also wanted to assess how their initial views towards obstacles to participation in the questionnaire survey, reflected their actual self-reported response rate.

Self-reported response rate amongst those who saw no obstacles to participation, was compared with the response rate of those who did see obstacles. To assess if those who could not be reached in the initial telephone survey, had different response rates and views, an equal number of those who were unreachable in the initial telephone interview was included in the second telephone interview for comparison.

After excluding all men who had died during the five- year time period between telephone interviews, 20 widowers were randomly selected from each group: A) Those who had participated in the initial telephone interview and reported no obstacles for participation in the questionnaire survey (n = 20); B) Those who had reported obstacles in the initial telephone interview (n = 20), and C) Those who were unreachable in the initial telephone interview (n = 20). This totals 60 widowers.

The second telephone survey took place from October 27 to November 11, 2007. As before, traditional hours for lunch and dinner were respected, and no calls were made after 21:00 hours. Each telephone call included an introduction of researcher as before, and an explanation of why the contact was being made. The widower was also informed that he was under no obligation to participate in this follow-up survey. The interviews were semi-structured as in the previous telephone study, and the interviews lasted 10-15 minutes.

## Results

No widower meeting the inclusion criteria was younger than 30 years, effectively defining the study population as 346 widowers born 1924-1969 (aged 30-75 years) alive at the time of the interview. Of the 346 identified widowers in the original study base, 86% (296/346) could be reached by telephone (Table 1), and all of those (100%) agreed to participate in the telephone survey. Of the sixty-one widowers that could not be reached by phone, 11 were deceased, and 50 could not be located (Table 1). The response rate in the following epidemiological questionnaire survey was 62% (216/346). Due to preliminary work for this study, it had already been more than one year since any widower's loss, by the time of our initial contact.

**Table 1:** Population characteristics and proportion of widowers in the first telephone survey identifying obstacles to participation in an upcoming questionnaire survey on bereavement.

		Age group of participants (widowers)				Years of grief following loss of spouse		
	All	30-49	50-59	60-69	70-75	2nd year	3rd year	4th year
Number of widowers in the original study base	**n = 346**	**n = 31**	**n = 76**	**n = 119**	**n = 120**	**n = 113**	**n = 117**	**n = 116**
Unreachable by phone/could not be located - % (n/N)	14% (50/346)	23% (7/31)	21% (17/76)	18% (21/119)	5% (6/120)	19% (22/113)	10% (12/117)	14% (16/116)
**Total number who could be reached**- % (n)	**n = 296** 86%	**n = 24** 77%	**n = 60** 79%	**n = 98** 82%	**n = 114** 95%	**n = 91** 81%	**n = 105** 90%	**n = 100** 86%
**Proportion of widowers identifying obstacles to participation **- % (n/N)	**55%** (163/296)	**33%** (8/24)	**48%** (29/60)	**57%** (56/98)	**61%** (70/114)	**55%** (50/91)	**53%** (56/105)	**57%** (57/100)

Further results from that survey are presently being analyzed and will be published at a later date.

Of all 296 widowers participating in the first telephone survey, 133 (45%) stated that they did not see any obstacles to further participation, but 163 (55%) stated that they did see obstacles to participation in a questionnaire survey (Table 1). Of the younger widowers (aged 30-59), 56% (47/84) expressed no obstacles to participation, compared with 41% (86/212) of the older widowers (aged 60-75) (Table 1). The relative risk with 95% CI comparing the younger and the older widowers was 1.38 (1.1-1.8).

In the current analysis, no apparent response difference was noted between years of grief following loss (second, third and fourth years) and expression of obstacles against participation in a questionnaire survey (Table 1).

Of the 163 (55%) who did see obstacles to participation in the upcoming questionnaire survey (in the first telephone survey), 37 men (23%) did not identify any particular obstacle. Of the 126 (77%) who did identify a particular obstacle, roughly a third (54/163) stated that "they felt bad" or that it would be "a painful experience" or that they felt "uncomfortable" talking about their grief. Twenty nine (18%) stated that their grief was "a private matter". Ten (6%) stated they did not want to be "stuck with their grief." Fifteen (9%) deemed it "too late" to talk about their grief or that they "wanted to look toward their future". Eighteen men (11%) stated "other reasons", including responses like: "it's too early to talk about it", and "I have started another relationship - don't want complications."

All those who could not be reached in the initial survey, received a message from the research group to call back, but none complied.

### The second telephone survey

All 60 widowers (100%) randomly selected to participate in the second telephone survey could be reached and all agreed to participate in the interview when contacted.

Of those who, in the first telephone survey, initially had not seen any obstacles to participation in the questionnaire survey, 95% reported they had responded to the questionnaire (Table 2). The corresponding percentage for those who had identified obstacles and for those who could not be reached in the initial survey was 30% for each group (Table 2). The relative risk with 95% CI was 3.2 (2.0-5.1) for the comparison between those seeing no obstacles versus others.

**Table 2:** Second telephone survey: Did you participate in the questionnaire survey?

	Saw no obstacles in the initial survey	Identified obstacles in the initial survey	Could not be reached in the initial survey
Responded to questionnaire.	95% (19/20)	30% (6/20)	30% (6/20)

Of the widowers, who initially had seen no obstacles, all stated they would participate in a bereavement survey today (Table 3). The corresponding percentage for those who had initially identified obstacles, indicated that they would participate, was 60%. Furthermore, 55% of those who could not be reached in the initial survey were now inclined to participate (Table 3). Thus, the majority of the initially negative and unreachable widowers stated five years later they would now participate in a bereavement survey. However, in the randomly selected sample included in the second telephone survey, 40% (8/20) of those who could see obstacles, and 45% (9/20) of the initially unreachable widowers, continued to be negative towards participation five years later (Table 3).

**Table 3:** Answers from the second telephone survey: If you were asked to participate in a bereavement survey today, would you participate?

	Saw no obstacles in the initial survey	Identified obstacles in the initial survey	Could not be reached in the initial survey
Yes, would participate	100% (20/20)	60% (12/20)	55% (11/20)
No, would not participate	0% (0/20)	35% (7/20)	20% (4/20)
Uncertain	0% (0/20)	5% (1/20)	25% (5/20)

## Discussion

All widowers initially contacted by telephone were willing to participate in the telephone interview and discuss possible obstacles to participation in a questionnaire survey. However, approximately half of them identified obstacles regarding participation in the impending questionnaire survey. The second telephone survey five years later revealed those initially seeing no obstacles to participation in the questionnaire survey were significantly more likely to report they had participated. The reported response rate amongst those who could not be reached in the initial telephone survey was similar to the reported response rate for those who could be reached, but identified obstacles.

These results indicate our original concerns may have been justified, regarding emotional obstacles affecting participation. Personal contact with participants prior to sending out a questionnaire may have enhanced response rate, since many of the men who initially saw obstacles in the telephone survey actually participated in the questionnaire survey. But we may also have been able to avoid unnecessary difficulties amongst at least some of those widowers who by receiving information about the questionnaire beforehand didn't open the envelope, and weren't exposed to a questionnaire they saw obstacles to responding.

All those who could not be reached in the initial survey, received a message from the research group to call back. The fact they did not call back, could possibly indicate that they saw obstacles, but other possibilities could be that they were away from home for an extended period, and/or may not have received the message.

The prevalence of obstacles in the initial telephone survey was not related to the time elapsed since the wives' deaths (Table 1). Perhaps the time-frame of the present study (second to fourth year since loss) was too short to capture the effect of time on bereavement. However, in the second telephone survey, five years later, those widowers who had seen obstacles were more willing to participate than before. The fact that a higher proportion saw no obstacles to participation in the second telephone survey, might indicate that time exceeding four years may also be a critical threshold for willingness to participate in studies of this kind.

However, it is also possible that exposure to the questionnaire may have decreased initial negative feelings towards the idea of answering a questionnaire on bereavement. Yet, of those randomly selected for follow- up five years later, 25%continued to express negative feelings or ambivalence towards participation (Table 3).

Younger widowers were more likely to see no obstacles to participation in the survey than older widowers (Table 1). One possible explanation may be that younger generations of Icelandic men may find it easier to confront and discuss emotionally taxing feelings. This would be consistent with studies from Sweden, which reported prevalence of emotional isolation among middle-aged and elderly men [24].

Comparison of our results with other studies in the scientific literature is problematic, since this is the first study to include a telephone survey before and after sending a questionnaire on male bereavement. Also, all available studies assessing the psychological impact of a questionnaire on participants are based on questions that are integrated into the questionnaire itself, and thus only represent those responding to the questionnaire [14-20].

It is always a delicate matter to approach people who have experienced bereavement, for the purpose of research. It has been argued that it may be unethical *not *to conduct research on bereavement services, since research is the only way to assess if a particular service has a positive or negative effect on the mourner [17]. However, the possibility that researchers may cause distress and harm needs to be weighed against possible benefits of gaining new knowledge and improving care of the bereaved [14,18-20,25]. Either way, researchers are responsible for what arises as a result of their research.

In spite of frequent doubts on ethical grounds regarding bereavement studies, many of the published data assessing questionnaire impact on participants has indicated a positive effect on the participants. In one study of bereaved parents who had lost a child from cancer, two-thirds of the parents perceived an inquiry into their child's death as having had a positive effect on them, four to nine years after their loss [15]. In another study on parents who had lost their children, it was reported that, in spite of the fact that participation in the study was painful to parents, it was perceived as important to the parents to tell their story to a respectful, empathetic, and informed researcher [16]. A study comprising a representative sample of Swedish widowers who lost their wife to cancer, showed that almost 90% of the widowers found the study valuable, and only 7% reported strong negative effects arising from it [17]. But emotional disclosure may not be as beneficial as was thought earlier [20,21], and the role of social support in protecting the bereaved against the impact of bereavement, and helping them to cope with the loss, are inconsistent [22].

In our study, the increased willingness over time to participate in future bereavement studies indicates a majority of the participants possibly found their participation in the telephone and questionnaire surveys was a more positive experience than a negative one. Other influencing factors cannot be excluded. It remains to be seen in future surveys if widows share the same obstacles as widowers regarding participation in such studies. Research on gender differences indicates that women may be more open regarding the sharing of emotionally taxing feelings and more willing to accept assistance in crisis situations [24,26].

*In summary*, our results indicate that a majority of widowers are willing to participate in bereavement research after receiving initial information concerning the content and aims of the study.

The resistance against participation in bereavement studies may decrease in the future, since younger widowers reported fewer obstacles, which may indicate a generation trend towards emotional openness? However, future bereavement research on middle-aged and elderly men needs to recognize that a substantial proportion of men feel reluctant to participate in this kind of study during the first four years of bereavement.

## Conclusions

In our study, a majority of the Icelandic bereaved male population identified obstacles to participation in a questionnaire survey regarding bereavement. Despite this, all those who could be reached by phone were willing to participate in the telephone survey. This indicates a strong interest in the subject. Also, the response rate to the epidemiological questionnaire survey following the telephone interview was 62% (216/346). However, approximately one third of the widowers were initially negative regarding participation and remained negative throughout the study period.

## Competing interests

The authors declare that they have no competing interests.

## Authors' contributions

ARH supervised the study design, analysis and writing of the paper. BS identified all subjects in the study base, constructed and conducted all telephone interviews, worked on the analysis and the writing of the paper, and collaborated with the Icelandic National Registry. The interpretation of data was done jointly by ARH and BS. All authors read and approved the final manuscript.

## Pre-publication history

The pre-publication history for this paper can be accessed here:

http://www.biomedcentral.com/1472-684X/9/7/prepub
